# The Substrates of Nonsense-Mediated mRNA Decay in *Caenorhabditis elegans*

**DOI:** 10.1534/g3.117.300254

**Published:** 2017-11-09

**Authors:** Virginia S. Muir, Audrey P. Gasch, Philip Anderson

**Affiliations:** Laboratory of Genetics, University of Wisconsin-Madison, Wisconsin 53706

**Keywords:** Nonsense-mediated mRNA decay (NMD), UPF1/SMG-2, RNA-Seq, *C. elegans*, post-transcriptional gene regulation

## Abstract

Nonsense-mediated mRNA decay (NMD) is a conserved pathway that strongly influences eukaryotic gene expression. Inactivating or inhibiting NMD affects the abundance of a substantial fraction of the transcriptome in numerous species. Transcripts whose abundance is altered in NMD-deficient cells may represent either direct substrates of NMD or indirect effects of inhibiting NMD. We present a genome-wide investigation of the direct substrates of NMD in *Caenorhabditis elegans*. Our goals were (i) to identify mRNA substrates of NMD and (ii) to distinguish those mRNAs from others whose abundance is indirectly influenced by the absence of NMD. We previously demonstrated that Upf1p/SMG-2, the central effector of NMD in all studied eukaryotes, preferentially associates with mRNAs that contain premature translation termination codons. We used this preferential association to distinguish direct from indirect effects by coupling immunopurification of Upf1/SMG-2 with high-throughput mRNA sequencing of NMD-deficient mutants and NMD-proficient controls. We identify 680 substrates of NMD, 171 of which contain novel spliced forms that (i) include sequences of annotated introns and (ii) have not been previously documented in the *C. elegans* transcriptome. NMD degrades unproductively spliced mRNAs with sufficient efficiency in NMD-proficient strains that such mRNAs were not previously known. Two classes of genes are enriched among the identified NMD substrates: (i) mRNAs of expressed pseudogenes and (ii) mRNAs of gene families whose gene number has recently expanded in the *C. elegans* genome. Our results identify novel NMD substrates and provide a context for understanding NMD’s role in normal gene expression and genome evolution.

Nonsense-mediated mRNA decay (NMD) is a post-transcriptional surveillance system that influences gene expression in eukaryotes by recognizing and degrading transcripts containing premature translation termination codons (PTCs) [reviewed in Schweingruber *et al.* (2013) and Lykke-Andersen and Jensen (2015)]. NMD contributes to the fidelity and precision of gene expression by eliminating aberrant mRNAs that, for varying reasons, are not translatable through the normal open reading frame. Previously described NMD substrates include unproductively spliced mRNAs (Morrison *et al.* 1997; Mitrovich and Anderson 2000; Lewis *et al.* 2003), mRNAs of expressed pseudogenes (Mitrovich and Anderson 2005), mRNAs of unproductively rearranged T-cell receptor and immunoglobulin genes (Gudikote and Wilkinson 2002), mRNAs with long 3′-untranslated regions (Kebaara and Atkin 2009) or upstream open reading frames (Mendell *et al.* 2004), mRNAs of snoRNA host genes (Lykke-Andersen *et al.* 2014), and mRNAs of transposons (Mendell *et al.* 2004) and viruses (Balistreri *et al.* 2014; Garcia *et al.* 2014). NMD was first discovered by its effects on β-globin mRNA of patients with β^0^-thalassemia (Baserga and Benz 1988), but it influences expression of a remarkably large fraction of the transcriptome in normal cells. Studies of many species demonstrate that between 4 and 25% of protein-coding genes exhibit altered expression when NMD is reduced or eliminated (He *et al.* 2003; Mendell *et al.* 2004; Rehwinkel *et al.* 2005; Metzstein and Krasnow 2006; Wittmann *et al.* 2006; Ramani *et al.* 2009; Yepiskoposyan *et al.* 2011; Rayson *et al.* 2012). Messenger RNAs with elevated abundance in NMD-deficient cells encode proteins involved in a wide variety of cellular processes, including RNA-processing (Ramani *et al.* 2009), translation (He *et al.* 2003; Rehwinkel *et al.* 2006), intracellular transport (He *et al.* 2003), amino acid metabolism (Mendell *et al.* 2004), DNA repair (Rehwinkel *et al.* 2006), and cell membrane dynamics (Guan *et al.* 2006).

A persistent complication of many such studies is that altered expression of any single gene in NMD-deficient cells could be due either to a direct effect, in which mRNAs of the affected gene are substrates of NMD, or to indirect effects, in which altered expression of the many substrates of NMD have indirect consequences on expression of other genes. Indeed, previous studies suggest that only a subset of mRNAs whose abundance is altered in NMD-deficient cells are NMD substrates (He *et al.* 2003; Mendell *et al.* 2004; Rehwinkel *et al.* 2005; Guan *et al.* 2006; Metzstein and Krasnow 2006; Wittmann *et al.* 2006; Johansson *et al.* 2007; Ramani *et al.* 2009; Yepiskoposyan *et al.* 2011; Rayson *et al.* 2012; Tani *et al.* 2012; Hurt *et al.* 2013; Matia-Gonzalez *et al.* 2013; Chapin *et al.* 2014; Schmidt *et al.* 2015). One of three different strategies has generally been employed to establish whether a transcript is a direct substrate of NMD: (i) isolating RNAs that copurify with UPF1/SMG-2 (Johansson *et al.* 2007; Hurt *et al.* 2013; Matia-Gonzalez *et al.* 2013); (ii) measuring mRNA half-lives after inactivation or inhibition of NMD, followed by monitoring immediate *vs.* delayed effects on expression (Guan *et al.* 2006; Tani *et al.* 2012; Schmidt *et al.* 2015); and (iii) measuring changes in mRNA abundance following reactivation of NMD (Maderazo *et al.* 2003; Johansson *et al.* 2007; Chapin *et al.* 2014). Collectively, these studies demonstrate that ∼20–50% of transcripts whose expression is increased in NMD-deficient cells are direct NMD substrates (Guan *et al.* 2006; Tani *et al.* 2012; Matia-Gonzalez *et al.* 2013). Thus, NMD has significant genome-wide effects on gene expression in normal cells. Interestingly, a substantial number of mRNAs appear to be substrates of NMD, but their steady-state abundance is unaltered in NMD-deficient cells (Tani *et al.* 2012).

Conserved proteins required for NMD have been identified in all tested eukaryotes, underscoring the evolutionary significance of the pathway. Three genes first identified in yeast (*upf1*, *upf2*, and *upf3*) (Leeds *et al.* 1991, 1992) encode core components of the NMD machinery. *Caenorhabditis elegans* orthologs of *upf1*, *upf2*, and *upf3* are named *smg-2*, *smg-3*, and *smg-4*, respectively. Four additional *C. elegans* genes (*smg-1*, *smg-5*, *smg-6*, and *smg-7*) are not found in yeast, required for NMD in *C. elegans*, and conserved widely in both plants and animals, including humans (Pulak and Anderson 1993; Cali *et al.* 1999; Page *et al.* 1999; Aronoff *et al.* 2001; Anders *et al.* 2003; Grimson *et al.* 2004; Behm-Ansmant *et al.* 2007). NMD-deficient mutants of yeast and *C. elegans* have relatively mild phenotypes (Leeds *et al.* 1991; Pulak and Anderson 1993), but NMD is essential in other eukaryotes (Medghalchi *et al.* 2001; Rehwinkel *et al.* 2005; Arciga-Reyes *et al.* 2006; Azzalin and Lingner 2006; Metzstein and Krasnow 2006; Wittkopp *et al.* 2009).

Upf1/SMG-2 is the central regulator of NMD. It is an ATP-dependent helicase and the most highly conserved of the NMD proteins (Perlick *et al.* 1996; Culbertson and Leeds 2003). When tethered to mRNAs either artificially (Lykke-Andersen *et al.* 2000) or naturally, as in Staufen-mediated decay (SMD) (Kim *et al.* 2005), mammalian UPF1 is sufficient to elicit mRNA instability. We previously demonstrated that *C. elegans* Upf1/SMG-2 (but not Upf2/SMG-3 or Upf3/SMG-4) is strongly and/or persistently bound to PTC-containing mRNAs (Johns *et al.* 2007). Such marking of mRNAs by Upf1/SMG-2 is especially pronounced in *smg-1*(−) and *smg-5*(−) mutants, both of which block NMD at a point after PTC discrimination but before mRNA degradation. SMG-1 is the Upf1/SMG-2 kinase (Yamashita *et al.* 2001; Grimson *et al.* 2004), and SMG-5 facilitates Upf1/SMG-2 dephosphorylation by PP2A (Page *et al.* 1999; Anders *et al.* 2003; Chiu *et al.* 2003). Phosphorylation of Upf1/SMG-2 occurs after PTC discrimination and promotes late-stage NMD steps, including the recycling of NMD components for future cycles of NMD (Lykke-Andersen *et al.* 2000; Durand *et al.* 2016).

We capitalized on the association of Upf1/SMG-2 with PTC-containing mRNAs to identify the direct substrates of NMD in *C. elegans*. Such mRNAs are expected to (i) increase in abundance in NMD-deficient mutants when compared with NMD-proficient controls, (ii) copurify with Upf1/SMG-2 from *smg-1*(−) mutants, and (iii) not co-purify with Upf1/SMG-2 in mock purifications from *smg-1*(−) *smg-2*(−) double-mutant controls. Our results identify new direct targets of NMD and provide new insights into the structure and expression of the *C. elegans* genome.

## Materials and Methods

### Nematode strains and growth

Strains used in this study were: N2 variety Bristol (wild type), TR2602 [*smg-1*(*r910*)], and TR2605 [*smg-1*(*r910*) *smg-2*(*r915*)]. None of the strains used contains the endogenous RNAi-related mutator allele *mut-16*(*mg461*), which is present in N2 variety Bristol laboratory stocks of many *C. elegans* laboratories (Zhang *et al.* 2011). We generated synchronized strains by treating gravid adults with a 5.25% hypochlorite solution (Stiernagle 2006). Embryos were grown to L4 stage in S-media at 20°.

### SMG-2 immunoprecipitation

We used the anti–SMG-2 polyclonal antibody described by Page *et al.* (1999) and a modified IP protocol described by Johns *et al.* (2007). Synchronously grown L4 animals were frozen in immunoprecipitation (IP) lysis buffer [20 mM MOPS (pH 7.2), 100 mM NaCl, 0.01% NP-40, protease inhibitor cocktail (Sigma), 1.46 µM pepstatin A (Sigma), 40 U/ml RNase OUT, and 10 mM PMSF]. Cells were lysed by sonication with 7 × 5 sec pulses, centrifuged at 14,000 × *g* for 25 min, and the supernatant fraction was retained. Samples were held at 4° through all IP and RNA extraction steps. Bradford assays measured protein concentrations of the extracts, which were diluted to 2 mg/ml in IP lysis buffer. An aliquot of this total RNA was preserved for sequence analysis, while a second aliquot was incubated with SMG-2 antibody coupled to Dynabead Protein G (Life Technologies), as previously described (Page *et al.* 1999). Precipitated proteins were collected on a magnet and washed six times with IP wash buffer [20 mM MOPS (pH 7.2), 100 mM NaCl, 0.02% NP-40]. Following RNA extraction with TRIzol (Life Technologies), input samples were DNaseI treated. IP samples exhibited no genomic DNA contamination via PCR and were not subjected to DNase treatment. A subsequent round of phenol chloroform extraction and ethanol precipitations further diminished contaminants prior to RNA sequencing (RNA-seq). Three independent, identically prepared samples with and without IP were submitted for sequencing as described below.

### RNA-seq

Poly(A) selection, cDNA library construction using the non-strand-specific Illumina TruSeq kit, and next-generation sequencing using an Illumina HiSeq 2000 platform were performed by the University of Wisconsin Biotechnology Center DNA Sequence Facility. Approximately 500 ng of RNA was used for library construction of samples without IP, and the entirety of submitted RNA (45–228 ng per negative control IP sample; 263–945 ng per experimental IP sample) was used for library construction of IP samples. Single-end reads were subjected to a mild quality trim using Trimmomatic (Bolger *et al.* 2014), and were aligned to the WBcel235 genome using Tophat2 (Kim *et al.* 2013).

### Bioinformatic analysis

Following alignment, both gene and intron counts were compiled using htseq-count (Anders *et al.* 2015). Features with fewer than three counts per million reads were excluded from downstream analysis. A reference intron-only annotation file was generated by merging each gene’s intersecting exons, as defined by the Ensembl GTF available through iGenomes, and using the intervening coordinates as intron coordinates. Differential expression analysis was performed using edgeR as previously described (Robinson *et al.* 2010; Anders *et al.* 2013). Briefly, we performed pairwise comparisons between wild-type and combined NMD-deficient mutant samples and between IP samples and their paired samples without IP. Sequence data were visualized using Broad’s IGV (Robinson *et al.* 2011). GO term statistical overrepresentation and pathway analysis were performed relative to all expressed genes using PANTHER and Gorilla (Eden *et al.* 2009; Mi *et al.* 2013). For GO analysis, we used binomial enrichment tests and the Bonferroni method to correct for multiple testing. For gene family comparisons, we used hypergeometric enrichment tests and the Bonferroni method to account for the two gene families tested. For gene category comparisons, we used hypergeometric enrichment tests and the Bonferroni method to correct for the 11 gene classes annotated in Ensembl.

### RT-PCR and qRT-PCR validation

First-strand synthesis was performed on aliquots of all input and IP samples using random hexamers and SuperScript III reverse transcriptase (Life Technologies). Prior to high-throughput sequencing, IP quality was tested using primers on either side of a PTC-containing, alternatively spliced intron of *rpl-12* (Mitrovich and Anderson 2000). Products were separated on a 2% ethidium bromide-stained agarose gel and quantified using ImageJ.

Following high-throughput sequence analysis, qPCR validation was performed on samples without IP using primers for *B0495.8*, *F45D11.1*, *F53B2.8*, *fib-1*, *fbxa-33*, *linc-9*, *nhr-109*, *pho-11*, *pqn-70*, *tdp-1*, *R08E5.3*, *rpl-7a*, *rsp-2*, *rsp-6*, *Y39B6A.21*, *Y51A2D.13*, and *ZK970.7*. Validation of *SMG-2* association was performed via RIP-coupled qRT-PCR using primers for *fib-1*, *fbxa-33*, *nhr-109*, *pho-11*, and *rpl-7a*. Included introns discovered via differential intron expression were validated using RT-PCR for *dpy-7*, *deg-1*, *phy-2*, and *R06C1.4*. The primer sequences used are provided in Supplemental Material, File S2.

### Data availability

Strains are available upon request from the *Caenorhabditis* Genetics Center. Antibodies are available upon request from the corresponding author at the University of Wisconsin. File S1 contains a list of Class I – Class IV mRNAs (discussed below). File S2 contains detailed descriptions of Supplemental Materials and Methods and Supplemental Figures. All sequence and sequence count data are available in the NIH SRA and GEO databases under accession number GSE100929.

## Results

### Transcriptome changes of NMD-deficient mutants

**Figure 1:**
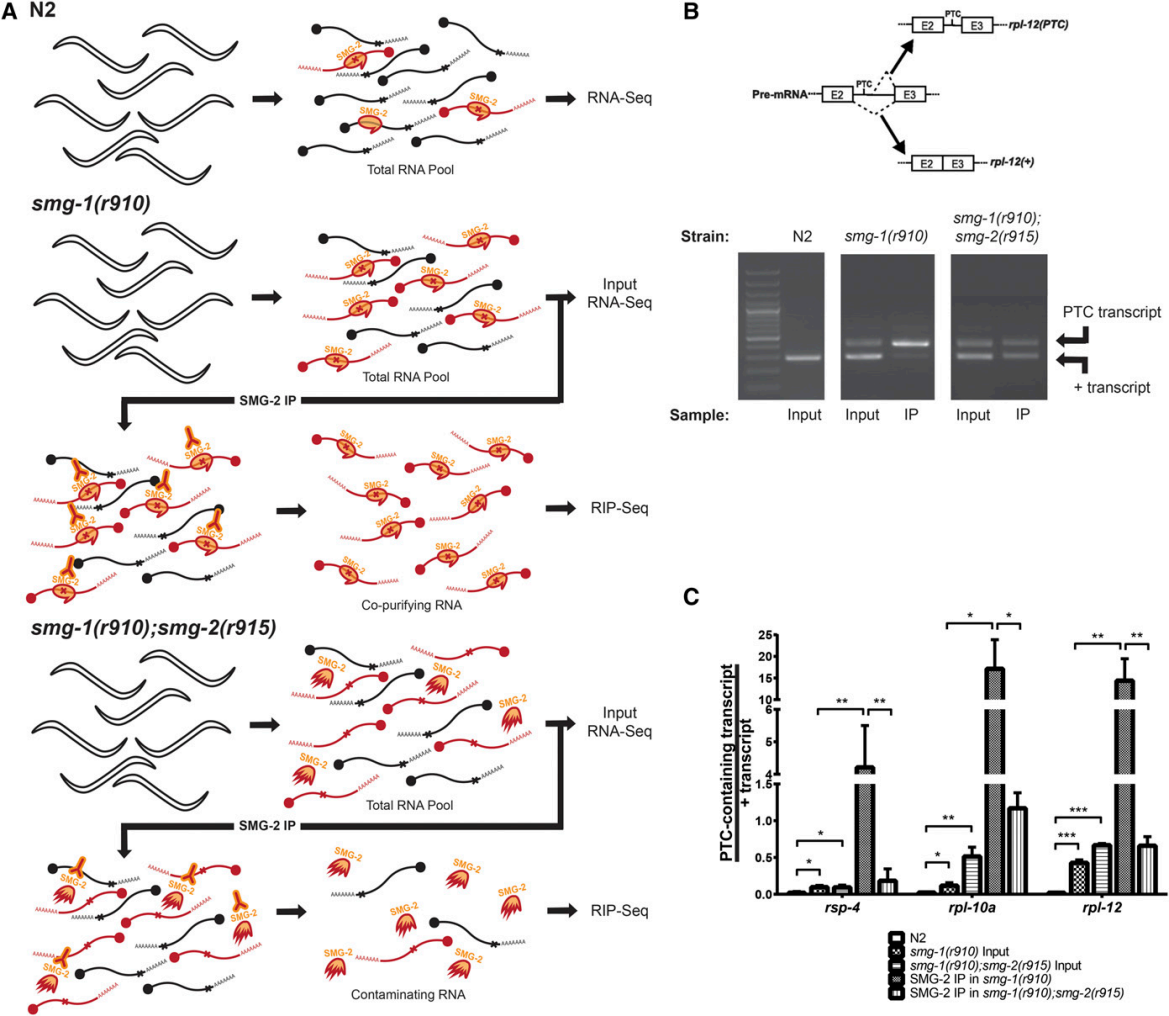
Validation of SMG-2’s preferential association with PTC-containing mRNAs. (A) Schematic diagram of RNA-seq and RIP-seq of SMG-2 IPs performed in this study. A portion of the total RNA from the *smg1*-(*r910*) and *smg1*-(*r910*) *smg-2*(*r915*) animals was sequenced for transcriptomic analysis (“input samples”) and the remainder was used for IPs. (B) Gene schematic showing accumulation of unproductively spliced *rpl-12* in NMD(−) animals (top), and validation PCRs detecting the *rpl-12* products in input and immunoprecipitated material from the two mutants. Both the schematic and gel are representative of other tested positive controls. (C) Quantification from PCR analysis validated the IP efficacy prior to high-throughput sequencing. Columns show the measured ratio of PTC-containing transcript to + transcripts for each gene. Error bars indicate SEM. *P*-values generated via Student’s *t*-test. Asterisks indicate significance: * *P* < 0.05, ** *P* < 0.01, *** *P* < 0.001.

Our strategy to identify genes negatively regulated by NMD and the direct substrates of NMD is shown in Figure 1A. Performing RNA-Seq of N2 (wild type), *smg-1*(*r910*), and an *smg-1*(*r910*) *smg-2*(*r915*) double mutant identified genes whose expression changes in NMD-deficient mutants. *smg-1*(*r910*) is a null allele resulting from Tc1 insertion at nucleotide position 17,105 of cosmid C48B6. It is strongly NMD defective, and SMG-1 protein of *r910* is not detected by Western blotting (Grimson *et al.* 2004). *smg-2*(*r915*) is a ∼1 kb internal deletion within *smg-2* (Page *et al.* 1999) that exhibits a strong NMD defect, as judged either by phenotypic suppression of *unc-54*(*r293*) (Pulak and Anderson 1993) or by stabilization of PTC-containing mRNAs (Page *et al.* 1999). Thus, *smg-2*(*r915*) is a functionally null allele. IP and sequencing of SMG-2-associated mRNAs in *smg-1*(*r910*) identified mRNAs that are associated with SMG-2. *Smg-1* mutations block NMD after PTC discrimination, after stable association of SMG-2 with PTC-containing mRNAs, but before mRNA degradation (Johns *et al.* 2007). Such SMG-2-associated mRNAs are highly enriched for those that contain PTCs. The principle advantage of identifying SMG-2-associated mRNAs in *smg-1*(*r910*) is that substrates of NMD are more abundant than in an NMD-proficient strain. Being more abundant, such mRNAs are easier to detect. Crude extracts were not treated with RNase prior to IP. Thus, intact or substantially intact RNAs were purified, and ensuing sequence analyses yielded information only on RNA abundance, not on precise locations of SMG-2 association with those RNAs. As a negative control for immunopurification of SMG-2-associated mRNAs, we simultaneously performed mock immunopurification of SMG-2 in a *smg-1*(*r910*) *smg-2*(*r915*) double mutant. For simplicity, *smg-1*(*r910*) and *smg-2*(*r915*) are hereafter termed *smg-1*(−) and *smg-2*(−), respectively.

**Figure 2:**
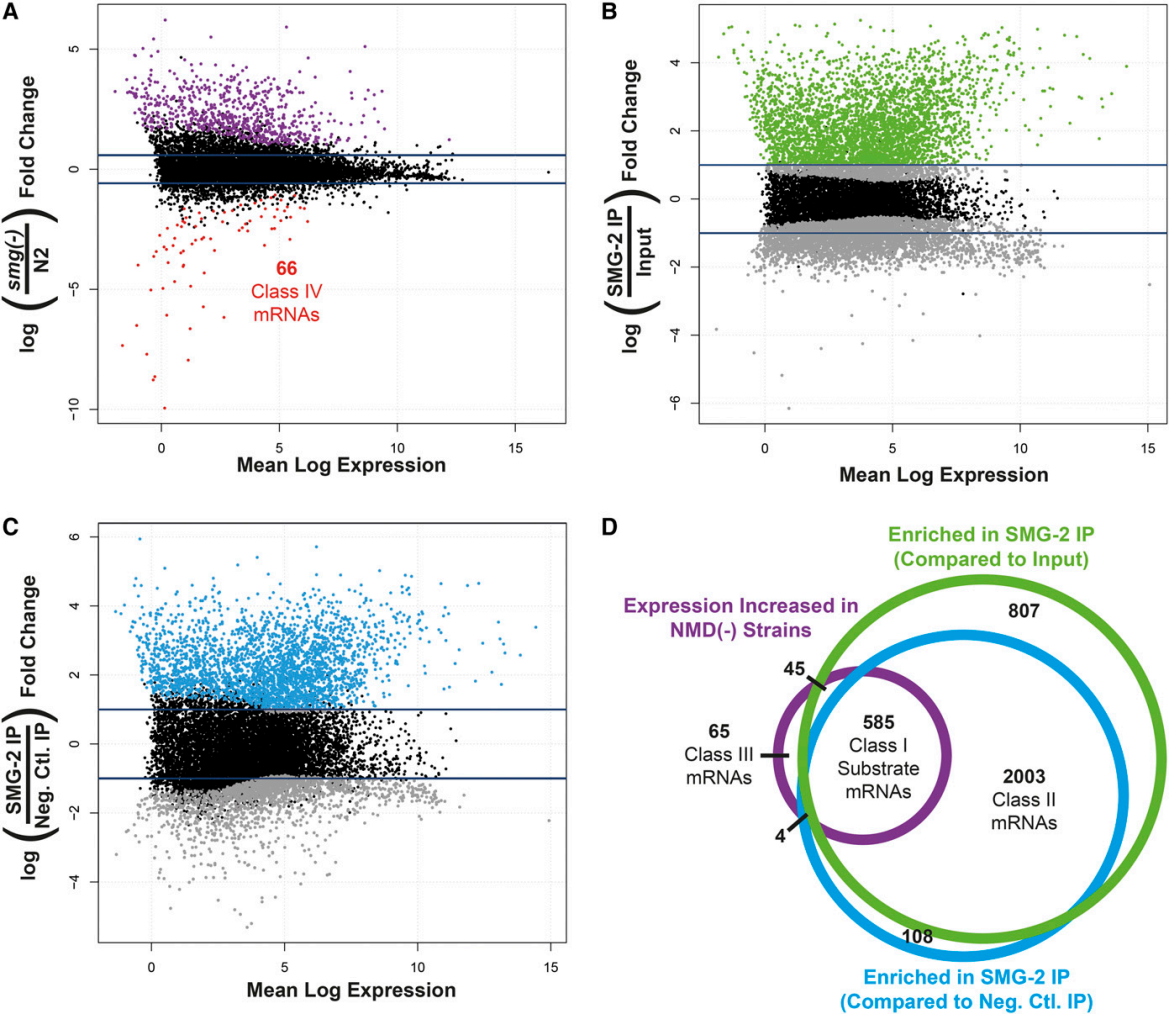
Determination of NMD direct substrates and indirect effects. (A) MA plots for all expressed genes. The *x*-axis depicts the mean normalized read count for each transcript across all samples, and the *y*-axis depicts log_2_(fold change) in gene expression in all NMD(−) samples [*smg-1*(*r910*) *smg-2*(*r915*) and *smg-1*(*r910*)] compared with wild type (N2). Each dot represents one gene, with purple and red dots representing genes with significantly upregulated and downregulated expression values (FDR < 0.05), respectively. Blue lines mark the 1.5-fold change threshold used in analysis. (B and C) MA plot of all genes (dots) with their average normalized read count on the *x*-axes and their relative enrichment comparing SMG-2 IPs performed in *smg-1*(*r910*) nematodes and either the *smg-1*(*r910*) input samples or the SMG-2 negative control IPs performed in *smg-1*(*r910*) *smg-2*(*r915*) nematodes on the *y*-axes, respectively. Significantly enriched transcripts (FDR < 0.05) that meet the twofold change threshold (blue lines) are shown in green (B) and blue (C) dots. (D) Venn diagram detailing overlap between transcripts exhibiting at least 1.5-fold increased abundance in *smg-1*(*r910*) and *smg-1*(*r910*) *smg-2*(*r915*) samples compared with N2, transcripts enriched at least twofold in an SMG-2 IP relative to its input sample, and transcripts enriched at least twofold in an SMG-2 IP relative to negative control IPs. Transcripts that meet all three conditions are classified as Class I NMD substrates. Transcripts that are reliably SMG-2 associated but that do not exhibit increased expression in an NMD(−) animal are deemed Class II, and genes with elevated expression in an NMD(−) sample but that never create mRNAs that are enriched in an SMG-2 IP are deemed Class III.

**Figure 3:**
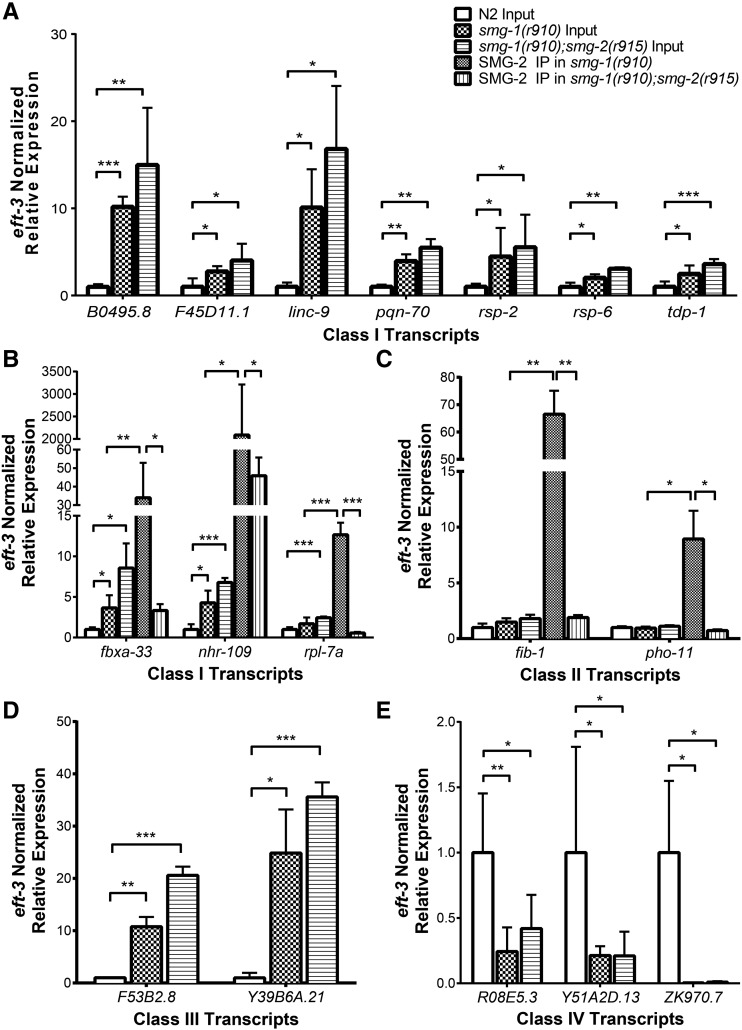
Validation of sequencing data. (A–E) qRT-PCR validation of expression changes and IP enrichments seen in high-throughput sequencing. Expression of (A and B) Class I, (C) Class II, (D) Class III, and (E) Class IV transcripts in input and IP samples (B and C only) was quantified using qRT-PCR on RNA preserved from the sequenced samples. All expression values were normalized to *eft-3* mRNA levels. Each column indicates the expression level relative to wild-type samples. Error bars indicate SEM for the three biological replicates, and statistical significance was calculated using Student’s *t*-test. Asterisks indicate significance: * *P* < 0.05, ** *P* < 0.01, ***P < 0.001.

We sequenced poly(A)-selected RNA extracted from populations of N2 (wild type), *smg-1*(−) single mutants, and *smg-1*(−) *smg-2*(−) double mutants synchronized at the L4 larval stage. Prior to sequencing, we removed a portion of each sample and processed it for SMG-2 IP (see below). We analyzed three independent biological replicates of each strain. Between 74 and 135 million sequence reads of each sample aligned to the *C. elegans* genome, representing 14,367 genomic features that met an expression minimum of at least three counts per million reads. Using edgeR to analyze differential expression, the abundance of 699 of the 14,367 genomic features (4.8%) increased when NMD-deficient mutants were compared as a combined set to N2 wild type (FDR < 0.05 and fold-change > 1.5; Figure 2A). The maximum upregulation was 79-fold in *smg-1*(−) and 69-fold in *smg-1*(−) *smg-2*(−) compared with N2 control samples. As expected, expression of the known PTC-containing mRNAs *rsp-2*, *rsp-6*, and *rpl-7a* (Johns *et al.* 2007) exhibited increased expression in NMD-defective strains (Figure 3, A and B). Expression of 74 genomic features was decreased in this analysis (FDR < 0.05 and fold-change > 1.5; Figure 2A). The maximum decrease was 5043-fold in *smg-1*(−) and 512-fold in *smg-1*(−) *smg-2*(−) compared with N2 control samples.

As a test of the accuracy of the above mRNA quantifications, we chose 12 previously unstudied genes whose expression is increased and three previously unstudied genes whose expression is decreased in NMD-deficient strains for further analysis. We designed gene-specific primers for each and quantified mRNAs by quantitative RT-PCR in triplicate in both NMD-deficient and N2 wild-type samples. For all 15 genes tested, both increased expression (Figure 3, A, B, and D) and decreased expression (Figure 3E) were confirmed by qRT-PCR.

### Identification of SMG-2-associated RNAs

Messenger RNAs that copurify with SMG-2 are strongly enriched for those that contain PTCs (Johns *et al.* 2007). To identify such RNAs, we purified mRNPs of *smg-1*(−) mutants by immunoprecipitating SMG-2 followed by RNA purification and cDNA sequencing (RIP-seq; Figure 1A). Such mRNAs are candidate substrates of NMD, but the IP pellets are undoubtedly contaminated with mRNAs whose presence is adventitious. To control for this, we performed parallel immunopurifications of SMG-2 from *smg-1*(−) *smg-2*(−) double mutants followed by RNA purification and cDNA sequencing. *smg-1*(−) *smg-2*(−) double mutants lack a functional SMG-2. This negative control was used to set a threshold for enrichment of mRNAs that copurify with SMG-2. We analyzed three biological replicate IPs of each strain. Between 42 and 75 million sequence reads of each IP sample aligned to the *C. elegans* genome, representing 14,124 genomic features that met an expression minimum of at least three counts per million reads. Prior to library preparation and high-throughput sequencing, we verified the efficacy of our IP protocols using mRNAs of the previously described NMD targets *rpl-12*, *rsp-4*, and *rpl-1* (Johns *et al.* 2007). Each of these genes expresses a PTC-containing, alternatively spliced mRNA that is stabilized in NMD-deficient strains and copurifies with immunoprecipitated SMG-2 (Figure 1, B and C).

We defined SMG-2-associated mRNAs as (i) those enriched at least twofold in the IP pellet of *smg-1*(−) compared with both the paired *smg-1*(−) sample without IP and the IP pellet of the negative control *smg-1*(−) *smg-2*(−) double mutant, and (ii) having an FDR < 0.05. We identified 2588 such mRNAs that are associated with SMG-2 using both of the comparisons listed above (Figure 2, B and C). Five of six known NMD substrates (*rpl-7a*, *rpl-12*, *rsp-2*, *rsp-4*, and *rsp-6*) (Johns *et al.* 2007) were among the 2588.

As an independent test of the accuracy of mRNA-SMG-2 associations, we chose five previously unstudied mRNAs from the list of 2588 and verified that each copurifies with SMG-2 by quantifying the mRNAs of IP pellets followed by quantitative RT-PCR (Figure 3, B and C). Such validations were performed in biological triplicates. In all five cases, the results obtained by IP followed by qRT-PCR confirmed those obtained by RIP-seq.

### A combined analysis of NMD-affected expression and SMG-2-associated mRNAs

As described above, sequencing the transcriptomes of NMD-proficient and NMD-deficient strains identified 773 mRNAs whose expression is influenced by NMD (699 increased and 74 decreased in NMD-deficient strains). Comparison of SMG-2 RIP-seq in *smg-1*(−) strains to the paired *smg-1*(−) input RNA-seq and negative control RIP-seq in *smg-1*(−) *smg-2*(−) strains identified 2588 RNAs that are SMG-2-associated. Combining these analyses identifies four relevant classes of genes, which we term Classes I–IV (Figure 2D and File S1 and below).

Expression of 585 Class I genes increases in NMD-deficient mutants, and those mRNAs copurify with SMG-2. Expression of 2003 Class II genes is unaltered in NMD-deficient mutants, but those mRNAs copurify with SMG-2. Expression of 65 Class III genes increases, and expression of 66 Class IV genes decreases in NMD-deficient mutants, but neither of those mRNAs copurify with SMG-2. Our interpretations of these classes are discussed below.

The 585 Class I mRNAs represent a high-confidence list of the direct substrates of NMD. Their abundance increases in NMD-deficient strains, and such mRNAs copurify with SMG-2. A total of 441 of the 585 Class I mRNAs derive from annotated, functional protein-coding genes, representing 2.9% of the estimated 15,000 such genes expressed at the L4 larval stage (Spencer *et al.* 2011). Four of these protein-coding genes are snoRNA hosts. An additional 131 Class I mRNAs (22% of total Class I mRNAs) derive from expressed pseudogenes. Messenger RNAs of expressed pseudogenes contain PTCs and, if translated, are degraded by NMD (Mitrovich and Anderson 2005). Only a subset of pseudogenes is expected to be transcribed, and only a fraction of those are expected to be translated. *C. elegans* contains an estimated 1658 pseudogenes (Yates *et al.* 2016), but the proportion that are expressed is uncertain. Estimates range from 16 to 25% (Gerstein *et al.* 2010; Sisu *et al.* 2014). Our results suggest that an important function of NMD is to reduce or eliminate pseudogene expression. Twelve additional Class I RNAs derive from genes of noncoding RNAs (five lncRNAs, four ncRNAs, and one antisense-lncRNA, -snoRNA, and -miRNA). One Class I RNA derives from a transposon. Although expression of the 2003 Class II mRNAs is not significantly altered in NMD-deficient strains, at least 5% of Class II mRNAs are likely to be substrates of NMD (see below).

We interpret the 65 Class III mRNAs (expression increased; not associated with SMG-2) and the 66 Class IV mRNAs (expression decreased; not associated with SMG-2) to reflect the indirect effects of eliminating NMD. Together, Classes III and IV account for 16% of genes having altered expression in NMD-deficient mutants. This relatively small number may reflect aspects of the way in which we identified mRNAs that copurify with SMG-2 (see *Discussion*). We conclude that a remarkably high fraction (84%) of mRNAs whose expression is significantly increased in NMD-deficient mutants are direct substrates of NMD.

Several classes of genes are enriched among Class I mRNAs. As discussed above, 22% of Class I mRNAs derive from expressed pseudogenes, which is a very strong enrichment compared with all expressed genes (Bonferroni-corrected *P* = 2.0e-82, hypergeometric test; Figure S1A in File S2). Additionally, Class I mRNAs are enriched for genes encoding nuclear hormone receptors (*nhr*) and F-box A proteins (*fbxa*) (Figure S1A in File S2). Both of these multigene families have undergone a remarkable and recent expansion of gene number in *C. elegans* (Sluder *et al.* 1999; Kipreos and Pagano 2000; Haerty *et al.* 2008; Taubert *et al.* 2011). The *C. elegans* genome contains 390 F-box protein genes (compared with 43 in *Drosophila* and 71 in humans) and 284 *nhr* genes (compared with 21 in *Drosophila* and 48 in humans). We identified 36 of 222 F-box A genes (Bonferroni-corrected *P* = 4.7e-19) and 15 of 284 *nhr* genes (Bonferroni-corrected *P* = 1.5e-2) as Class I substrates of NMD. The observation that NMD substrates are enriched for members of gene families whose gene number has recently expanded is provocative, particularly since many *fbxa* and *nhr* genes are Ensembl-annotated pseudogenes (see *Discussion*).

GOrilla functional annotation enrichment and PANTHER statistical overrepresentation demonstrate that Class I genes are enriched for nucleic acid binding (Bonferroni-corrected binomial test, *P* = 8.1e-04), mRNA splicing (Bonferroni-corrected *P* = 2.1e-02), and mRNA metabolic processes (Bonferroni-corrected *P* = 7.5e-03) (Figure S1B in File S2). Similarly, Class II genes are enriched for nucleic acid binding (Bonferroni-corrected *P* = 1.4e-04), mRNA splicing (Bonferroni-corrected *P* = 2.2e-02), and mRNA metabolic processes (Bonferroni-corrected *P* = 3.1e-04) (Figure S1C in File S2). Class III and IV genes (those indirectly affected by the NMD pathway) are enriched for factors involved in the immune, defense, and stress response systems (Bonferroni-corrected *P* < 0.01; Figure S1D in File S2).

### Class II mRNAs include alternatively spliced NMD substrates

Approximately 2000 Class II mRNAs copurify with SMG-2, but their expression is not significantly increased in NMD-deficient mutants (Figure 2D). Like Class I genes, Class II genes are enriched for nucleic acid binding, mRNA splicing, and mRNA metabolism and processing GO terms (see above), pseudogenes (162 genes, Bonferroni-corrected *P* = 5.3e-38, hypergeometric test), and nuclear hormone receptors (47 genes, Bonferroni-corrected *P* = 2.9e-5). The similarity between Class I and Class II genes suggests that many Class II mRNAs are, in fact, substrates of NMD.

Alternatively spliced mRNAs that contain PTCs are a well-established class of NMD substrates. We therefore examined Class II genes for those in which (i) the gene encodes multiple isoforms of mRNA due to alternative splicing; (ii) only one (or a subset) of the mRNA isoforms contains PTCs; (iii) PTC-containing mRNAs increase significantly in abundance in NMD-defective mutants; and (iv) the fraction of PTC-containing mRNAs is small, causing the total quantity of mRNA (PTC-free plus PTC-containing) to not meet our statistical threshold for increased expression.

**Figure 4:**
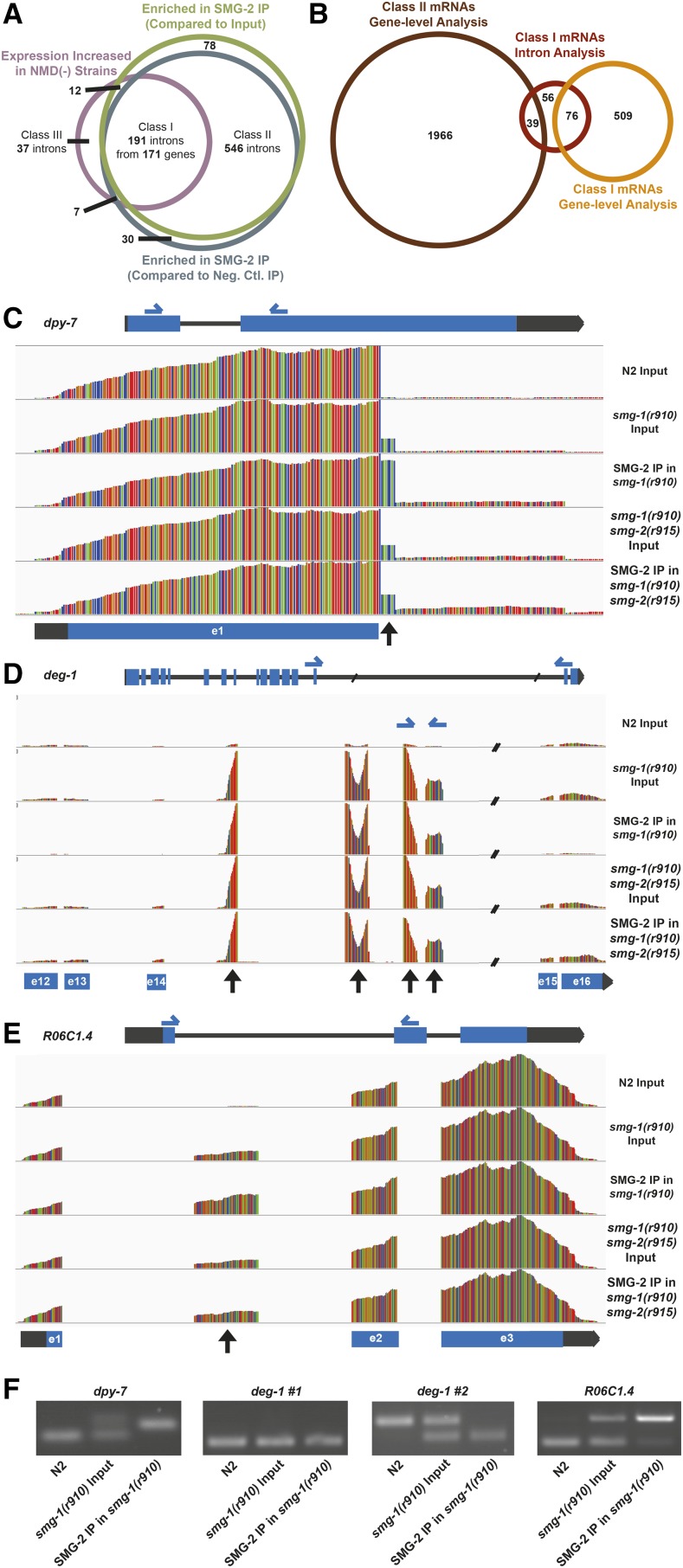
Differential expression of intronic sequence identifies more NMD substrates. (A) Venn diagram detailing overlap between introns exhibiting at least 1.5-fold increased expression in *smg-1*(*r910*) and *smg-1*(*r910*) *smg-2*(*r915*) samples compared with N2, introns enriched at least twofold in an SMG-2 IP relative to its input sample, and introns enriched at least twofold in an SMG-2 IP relative to negative control IPs. Class I introns meet all three conditions. Class II introns are reliably SMG-2-associated but do not exhibit increased expression in an NMD(−) animal, and Class III introns exhibit elevated expression in an NMD(−) sample but are never enriched in an SMG-2 IP. (B) Venn diagram comparing genes containing Class I Introns (red) with Class I and Class II mRNAs identified in the gene-wide analysis (see Figure 2). Fifty-six genes with only Class I introns and 39 genes with both Class I introns and Class II mRNAs were added to the list of high-confidence NMD substrates. (C–E) Relative sequencing coverage shown by IGV plots for three genes containing Class I introns: *dpy-7*, *deg-1*, and *R06C1.4*. Gene diagrams are shown below coverage plots. Bar height indicates relative sequencing coverage. Primer locations for PCR validation are shown as blue half arrows. Black arrows indicate intronic regions that demonstrate increased expression in NMD(−) samples and enrichment in the SMG-2 IP. (F) PCR validation for Class I introns. Primers were designed to span expressed introns for *dpy-7*, *deg-1* #1, and *R06C1.4*. Upper bands for these genes indicate that a portion of the intron was included in the mature transcripts present in the *smg-1*(*r910*) input sample and enriched in the SMG-2 IP. *deg-1* #2 primers span the third and fourth expressed regions in the intron of *deg-1*. The smaller putative isoform shown by the lower band in this PCR coprecipitates with SMG-2 and is present in an NMD(−) sample.

We first established a database of all introns of all annotated *C. elegans* genes as described in *Materials and Methods*. We then repeated the bioinformatic analyses described above, counting sequence reads that fall within coordinates listed in the intron database instead of annotated coordinates of gene bodies. We identified 247 introns of 220 genes that exhibit at least 1.5-fold increased expression in NMD-deficient strains compared with the NMD-proficient N2 wild-type (FDR < 0.05) and 737 introns of 630 genes that were enriched at least twofold in the SMG-2 IP pellet of *smg-1*(−) mutants compared with both the *smg-1*(−) sample prior to IP and the IP pellet of *smg-1*(−) *smg-2*(−) double-mutant controls. A combined analysis of intron expression and copurification with SMG-2 identified 191 introns of 171 genes that met statistical criteria for Class I NMD substrates (Figure 4A). Of the 171 genes identified in this manner, 76 had been identified as Class I mRNAs and 39 as Class II mRNAs by our original gene body-level analysis (Figure 4B). A previously described NMD substrate, *rsp-4* (Johns *et al.* 2007), which was not originally identified as a Class I substrate in the gene-level analysis, was identified as a Class I substrate via intron-level analysis.

We additionally analyzed five previously unstudied genes whose introns satisfied our criteria for Class I mRNAs using the Broad Institute’s Integrative Genomics Viewer (Robinson *et al.* 2011), followed by confirmation with RT-PCR (Figure 4, C–F and Figure S2 in File S2). Regions of identified introns that satisfied the criteria for Class I NMD substrates were unambiguous. For example, alternative splicing of *dpy-7* pre-mRNA utilizes an alternative 5′ splice site at the end of exon 1 that is seven nucleotides downstream of the annotated splice site, within the previously annotated intron. This variant mRNA is only detectable in a *smg*(-) strain and is strongly enriched in the mRNAs that copurify with SMG-2. Similarly, an alternatively spliced isoform of *R06C1.4* mRNA includes a portion of the previously annotated first intron. Abundance of the alternatively spliced mRNA increases significantly in NMD-deficient mutants, and it copurifies with SMG-2. We identified a putative novel transcript in the NMD-deficient mutants between *deg-1* exons 14 and 15. Sequence reads aligning to four distinct areas of the intron are far more abundant than reads aligning to *deg-1* exons. Although PCR primers flanking the intron do not detect multiple isoforms, primers specific for the enriched intronic regions detect independent NMD-responsive RNAs. In total, we confirmed intronic sequences in *smg*(-) transcripts in four of the five NMD-affected introns that we analyzed (Figure 4, D and E and Figure S2 in File S2).

We therefore added the 95 mRNAs identified as Class I substrates by intron-level analysis to the previous list of 585 high-confidence of NMD substrates, yielding a total high-confidence list of 680 mRNAs. Inclusion of these 95 additional mRNAs as Class I substrates did not significantly alter the enrichment of pseudogenes, gene families, or functional pathways discussed above.

## Discussion

NMD regulates eukaryotic gene expression by identifying and degrading mRNAs containing PTCs. NMD was discovered by its effects on β-globin mRNA of patients with β^0^-thalassemia (Baserga and Benz 1988), but NMD affects expression of many normal (nonmutant) genes. By degrading aberrant mRNAs, NMD is a surveillance system that contributes to both the fidelity and precision of gene expression. Some NMD substrates likely result from errors of gene expression (for example, base misincorporation during transcription or use of inappropriate splice sites), but many others are purposeful aspects of regulated gene expression. For example, regulated alternative splicing and programmed translational frameshifting regulate the expression of many genes by eliciting NMD [reviewed in Lykke-Andersen and Jensen (2015)]. Previous investigations of several species demonstrate that expression of a significant fraction (4–25%) of total mRNAs increases when NMD is reduced or eliminated (He *et al.* 2003; Mendell *et al.* 2004; Rehwinkel *et al.* 2005; Metzstein and Krasnow 2006; Wittmann *et al.* 2006; Ramani *et al.* 2009; Yepiskoposyan *et al.* 2011; Rayson *et al.* 2012). Two issues persistently complicate such studies: (i) NMD is essential in many multicellular species, where its function can be reduced but not eliminated, and (ii) perturbing NMD has both direct and indirect effects on the abundance of mRNAs.

We describe an investigation designed to distinguish the direct substrates of NMD from the indirect effects of eliminating NMD in *C. elegans*, a species for which NMD is nonessential and can therefore be completely eliminated. We identified NMD direct substrates as those that meet two criteria: (i) their expression is increased in mutants that lack NMD, and (ii) they copurify with Upf1/SMG-2 from *smg-1*(−) mutants but not from *smg-1*(−) *smg-2*(−) double-mutant controls. We detected poly(A)^+^ mRNAs of 14,780 of the estimated 15,000 protein-coding genes expressed at the L4 larval stage. Expression of 699 (4.7%) of these genes was significantly increased in NMD-deficient mutants, a fraction that is low but within the range established by previous work in both *C. elegans* (Ramani *et al.* 2009) and other organisms (Mendell *et al.* 2004; Rehwinkel *et al.* 2005; Yepiskoposyan *et al.* 2011; Tani *et al.* 2012; Matia-Gonzalez *et al.* 2013; Chapin *et al.* 2014). A total of 84% of these genes (585 out of 699) proved to be direct substrates of NMD. The magnitude of indirect effects of eliminating NMD in *C. elegans* is remarkably low in our experiments, representing only 0.9% of total genes. Such indirect effects include the 65 Class III mRNAs (increased expression; not SMG-2-associated) and the 74 Class IV mRNAs (reduced expression; not SMG-2-associated).

The proportion of expression increases due to direct NMD effects in *C. elegans* is considerably higher than observed in other species. For example, approximately half of the mRNAs negatively regulated by NMD in yeast are direct effects (Guan *et al.* 2006; Johansson *et al.* 2007), and ∼1/4 in HeLa cells (Tani *et al.* 2012). The paucity of indirect effects in *C. elegans* could be a species-specific phenomenon, but our method of measuring SMG-2 copurification with target mRNAs may have contributed to improved accuracy of identifying direct effects. We measured such interactions by identifying mRNAs that copurify with SMG-2 in a *smg-1*(−) *smg-2*(*+*) genetic background. PTC-containing mRNAs are more abundant in *smg-1*(−) strains, and the association of SMG-2 with NMD substrates is stabilized in such mutants (Johns *et al.* 2007). *Smg-1*(−) mutations block NMD after PTC discrimination, after stable association of SMG-2 with PTC-containing mRNAs, but before mRNA degradation. Such effects make mRNA-SMG-2 interactions easier to detect. We further filtered out mRNAs that contaminate an immunopurification of SMG-using a *smg-1*(−) *smg-2*(−) double-mutant control.

Class II mRNAs are those that copurify with SMG-2 but whose expression is not significantly increased in NMD-deficient mutants. We identified 2003 such mRNAs, severalfold more than the 585 Class I direct substrates. Similar studies with other species consistently observe a large class of NMD substrates whose expression is not increased when NMD is reduced or eliminated (Johansson *et al.* 2007; Tani *et al.* 2012; Matia-Gonzalez *et al.* 2013; Chapin *et al.* 2014; Schmidt *et al.* 2015). Several phenomena likely contribute to the large number of Class II mRNAs:

1. Some Class II mRNAs are certainly *bona fide* NMD substrates, but their abundance does not increase sufficiently to meet statistical thresholds. For example, the 95 Class II mRNAs discussed above are NMD substrates owing to unproductive alternative splicing and retention of intron fragments. Expression of such alternatively spliced genes is not significantly increased because, in each case, the PTC-containing mRNAs are only a fraction of total mRNA of the gene. Additional examples of such genes are likely included among Class II mRNAs.
2. Upf1/SMG-2 participates in mRNA turnover pathways in addition to NMD. For example, Upf1 directly interacts with the RNA-binding protein Staufen 1 during SMD (reviewed in Park and Maquat (2013)). Upf1:Staufen interactions localize Upf1 to Staufen-bound mRNAs, whereupon Upf1 triggers decay (Kim *et al.* 2005; Gong *et al.* 2009). Additionally, Upf1 is recruited to histone mRNAs and is required for replication-dependent histone mRNA decay (Kaygun and Marzluff 2005). Upf1 triggers decay in each case, but the mRNP complexes and mechanisms of activating decay are distinct from those of NMD. SMG-1 is reported to improve the efficiency of SMD in mammalian adipogenesis (Cho *et al.* 2013), but neither SMD nor histone mRNA decay is known to require NMD factors beyond Upf1. Thus, some Class II mRNAs likely reflect functions of Upf1 in other degradation pathways.
3. Upf1/SMG-2 associates weakly or transiently with all mRNAs, but its associations with PTC-containing mRNAs are strong and/or persistent (Johns *et al.* 2007; Lee *et al.* 2015; Durand *et al.* 2016). Transient interactions of Upf1/SMG-2 with PTC-free mRNAs may have been captured by our RIP-seq protocols. Such interactions would be absent in the *smg-1*(−) *smg-2*(−) double mutant, which was our control for specificity of SMG-2 purification. Perhaps many Class II mRNAs are simply normal (PTC-free) mRNAs for which PTC discrimination occurs more slowly than average and, hence, SMG-2 remains associated with the mRNA for a longer period of time than average during normal translation termination.

The 680 high-confidence substrates of *C. elegans* NMD are strongly enriched for three classes of genes: pseudogenes, nuclear hormone receptors (*nhr*), and F-box A (*fbxa*) proteins. Fully 22% of NMD substrates derive from expressed pseudogenes. The *C. elegans* genome contains an estimated 1658 pseudogenes (Yates *et al.* 2016), although the fraction of these that is expressed is uncertain. Two previous studies of 911 and 1293 *C. elegans* pseudogenes estimated that 16 and 25%, respectively, were represented in the RNA pool (Gerstein *et al.* 2010; Sisu *et al.* 2014). By extrapolation, between ∼250 and ∼400 total expressed *C. elegans* pseudogenes are predicted, of which we identified 131 as NMD substrates. *Fbxa* and *nhr* genes are particularly notable, as both families have undergone a remarkable and recent expansion of gene number in *C. elegans* (Sluder *et al.* 1999; Kipreos and Pagano 2000; Haerty *et al.* 2008; Taubert *et al.* 2011). The *C. elegans* genome contains 390 F-box protein genes, as compared with 43 in *Drosophila* and 71 in humans. Similarly, *C. elegans* contains 284 *nhr* genes, as compared with 21 in *Drosophila* and 48 in humans. We identified mRNAs of 36 of 222 *fbxa* genes (Bonferroni-corrected *P* = 4.7e-19) and 15 of 284 *nhr* genes (Bonferroni-corrected *P* = 1.5e-2) as NMD substrates. Our sequence tags often do not distinguish whether such *fbxa* and *nhr* substrates derive from pseudogenes or functional genes, but we note that annotated pseudogenes include 36 members of the *fbxa* and 10 members of the *nhr* families.

Although NMD is highly-conserved in all eukaryotes analyzed to date, NMD targets are not generally conserved in distantly related species. Of the 680 genes we identified as high-confidence NMD substrates, 199 have orthologs in yeast (Johansson *et al.* 2007), flies (Chapin *et al.* 2014), or mice (Hurt *et al.* 2013). Only five of these 199 genes are NMD substrates in both *C. elegans* and at least one other species. *him-14*, *gpx-4*, and their orthologs are NMD substrates in yeast (Johansson *et al.* 2007) and nematodes, while *lipl-4*, *ugt-60*, and their orthologs are NMD substrates in *Drosophila* (Chapin *et al.* 2014). Messenger RNAs of only a single gene, *aard-21* and orthologs, are NMD substrates in nematodes, flies, and mice (Hurt *et al.* 2013; Chapin *et al.* 2014). The structural features of NMD substrates, including pseudogene origins, PTCs resulting from alternative splicing, long 3′-UTRs, upstream open reading frames, *etc*. are conserved widely among eukaryotes. Nonetheless, individual genes are not typically conserved as NMD substrates outside of Mammalia (Rehwinkel *et al.* 2006; De Lima Morais and Harrison 2010). The lack of evolutionarily conserved substrates highlights that a pathway such as NMD can play important roles in species-specific functions and organismal evolution.

The remarkable number of NMD substrates derived from expressed pseudogenes suggests an important role for NMD in genome evolution. Pseudogenes that derive from complete gene duplications or amplifications begin their existence as functionally redundant genes. Such genes accumulate spontaneous mutations, including nonsense and frameshift alleles that render their mRNAs sensitive to NMD. Such pseudogenes will likely be transcribed and translated until their promoters decay by mutation or they are silenced epigenetically. Expressed pseudogenes are not constrained to produce functional proteins, but they are constrained to avoid expressing disruptive proteins or protein fragments. Most pseudogenes evolve neutrally, but some exhibit unexpected evolutionary constraints (Balakirev and Ayala 2003). Translation of pseudogene mRNAs is potentially deleterious, as exemplified by the phenomenon of NMD-dependent dominance. Certain *C. elegans* nonsense alleles are recessive in NMD-proficient genetic backgrounds, but they are strongly dominant in NMD-deficient backgrounds (Pulak and Anderson 1993; Cali and Anderson 1998). We suggest that NMD protects organisms from such disruptive effects by degrading pseudogene mRNAs, thereby modifying phenotypes. Potentially deleterious nonsense or frameshift alleles are recessive (instead of dominant) as heterozygotes and less deleterious as homozygotes in NMD-proficient genetic backgrounds. This effect is analogous to the role of NMD in masking the dominant phenotypes of certain heritable diseases (Miller and Pearce 2014). NMD may allow expressed pseudogenes to evolve neutrally and to persist for a greater number of generations in a population. Their persistence increases opportunities for acquiring new functions, such as evolving to become long noncoding RNAs (Grander and Johnsson 2016) or contributing sequence variation to functional genes via gene conversion (Balakirev and Ayala 2003).

## Supplementary Material

Supplemental material is available online at www.g3journal.org/lookup/suppl/doi:10.1534/g3.117.300254/-/DC1.
