# Supplementary material for: The Substrates of Nonsense-Mediated mRNA Decay in Caenorhabditis elegans
Source: G3 (Bethesda). 2017 Nov 9;8(1):195–205. doi: 10.1534/g3.117.300254 (PMC5765348; doi:10.1534/g3.117.300254)
Supplement: Supplementary file 2 [file 195FileS2.docx]

Supplementary Materials

Primer Sequences


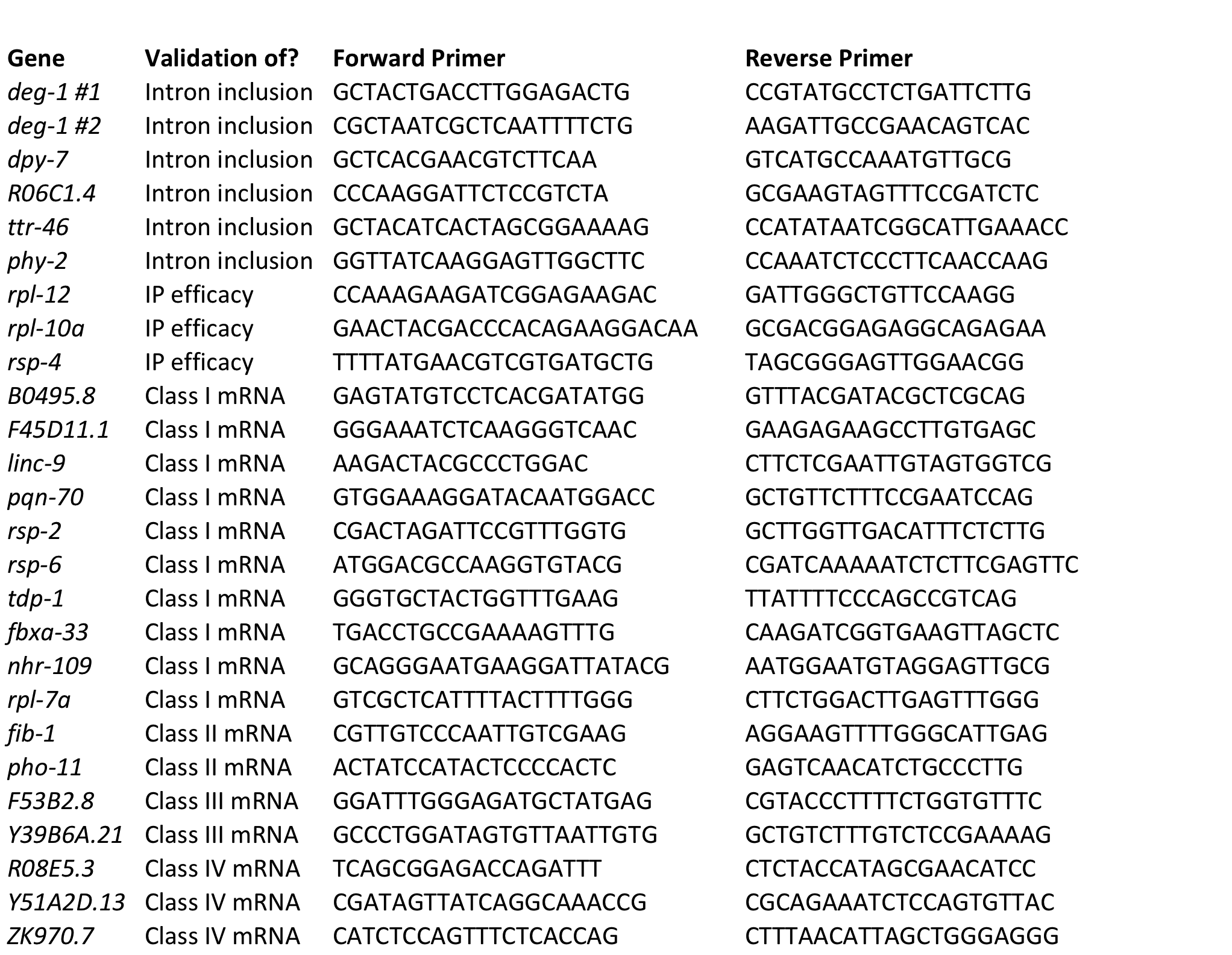


**File S1: Summary of Class I – Class IV RNAs**

File S1 contains a list of RNAs belonging to each of the classifications discussed in the manuscript. Gene IDs and log_2_ fold changes for each of the three major comparisons are provided.

**Figure S1: Statistical overrepresentation of gene classes among genes affected by NMD**


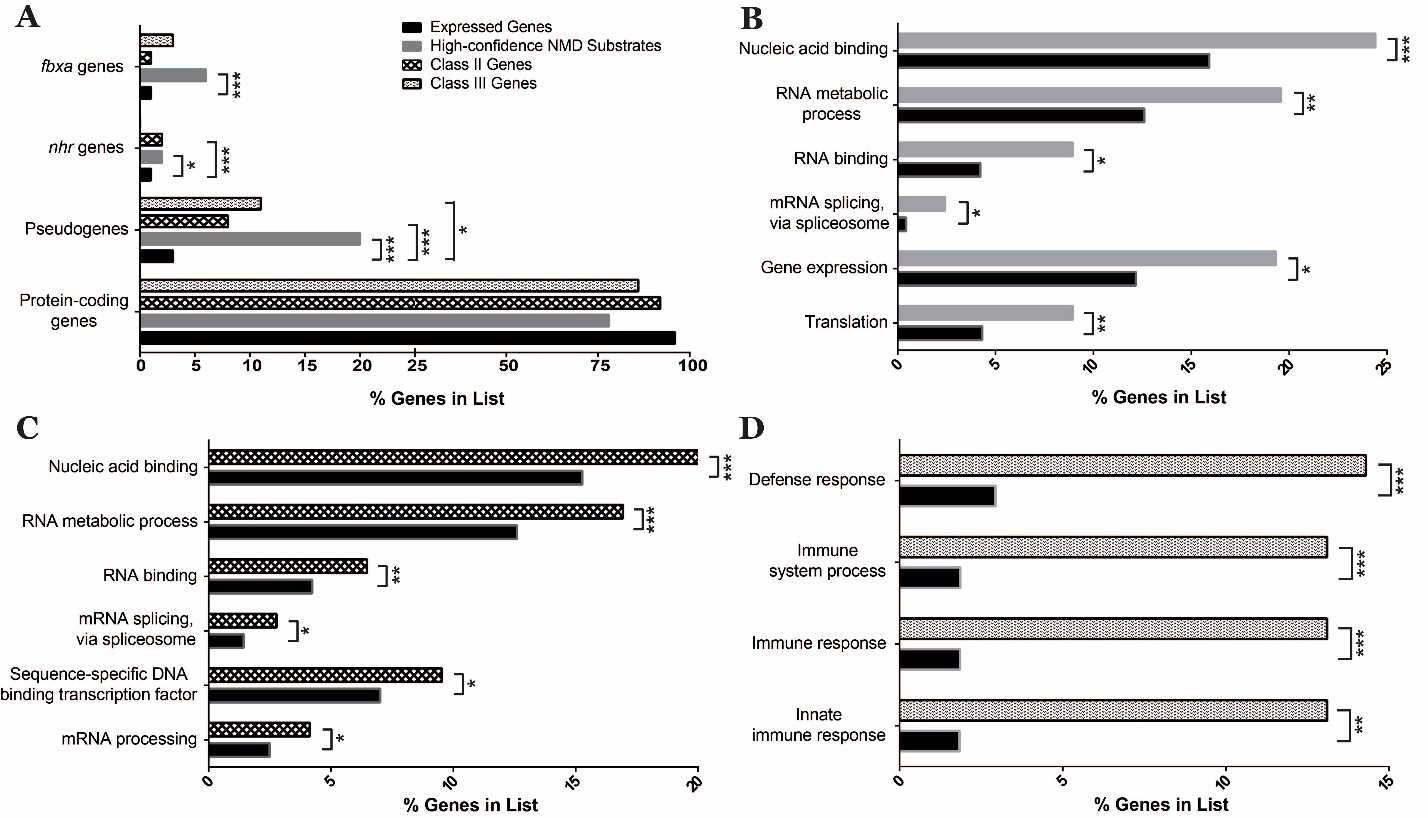


(A) Abundance of gene classes and gene types represented by Class I (“High-confidence NMD Substrates”), II, and III features compared to all expressed genes. P-values are derived from Bonferroni-corrected hypergeometric tests. (B-D) Abundance of GO terms found for (B) Class I, (C) Class II, and (D) Combined Indirect Effect (Class III & IV) lists, compared to expressed genes. P-values are derived from Bonferroni-corrected binomial tests, as performed by PANTHER’s Statistical Overrepresentation function. Asterisks indicate significance: * *p*<0.05, ** *p*<0.01, ****p*<0.001

**Figure S2: Differential expression of intronic sequence identifies more NMD substrates**


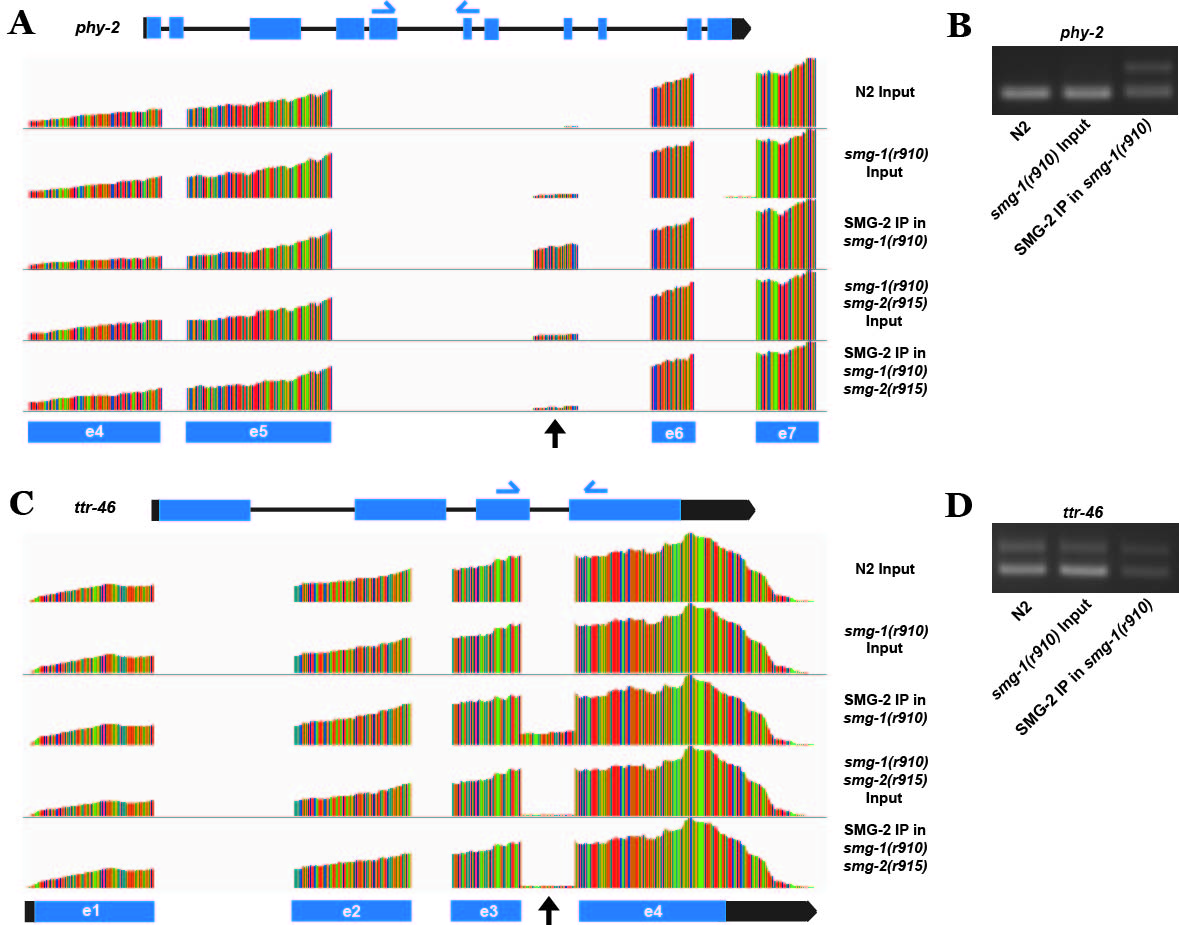


(A & C) Relative sequencing coverage shown by IGV plots for two additional genes containing Class I Introns: *phy-2* and *ttr-46*, respectively*.* Gene diagrams are shown below coverage plots. Colored bar height indicates relative sequencing coverage. Blue half arrows mark placement of primers for PCR validation. Black arrows indicate intronic regions that demonstrate increased expression in NMD(-) samples and enrichment in the SMG-2 IP. (B & D) PCR validation for Class I Introns. Primers were designed to span expressed introns for *phy-2* and *ttr-46*. Upper bands for these genes indicate that a portion of the intron was included in the mature transcripts present in the *smg-1(r910)* input sample and enriched in the SMG-2 IP.
